# Thyroid peroxidase antibodies do not impair the ovarian reserve in euthyroid women: a cross-sectional study

**DOI:** 10.1530/EC-25-0151

**Published:** 2025-08-25

**Authors:** Chongjuan Gu, Chaomin Yue, Ling Li, Jie Li, Chunting Wu, Yaojuan He

**Affiliations:** ^1^ Department of Obstetrics and Gynecology, Guangzhou Women and Children’s Medical Center, Guangzhou Medical University, Guangzhou, China; ^2^ Institute of Pediatrics, Guangzhou Women and Children’s Medical Center, Guangzhou Medical University, Guangzhou, China; ^3^ Department of Gynecology, Zhanjiang Central People’s Hospital, Zhanjiang, Guangdong, China

**Keywords:** ovarian reserve, anti-Müllerian hormone, thyroid function, thyroid peroxidase antibodies, autoimmune thyroid diseases

## Abstract

**Aim:**

This study aims to investigate whether thyroid peroxidase antibodies (TPOAb) affect the ovarian reserve using the anti-Müllerian hormone (AMH), basal follicle-stimulating hormone (bFSH), and estradiol (bE2) as markers.

**Methods:**

A large retrospective cross-sectional study was conducted, including women who visited our clinic between May 2016 and April 2024 and underwent same-day assessments of basal sex hormones, thyroid function, and AMH. Women with thyroid cancer, ovarian cancer, or clinical/subclinical hypo- or hyperthyroidism were excluded. Participants were stratified into TPOAb-positive and TPOAb-negative groups. Ovarian reserve markers and thyroid function were compared between the groups, and the impact of TPOAb positivity on ovarian reserve was analyzed.

**Results:**

Totally, 3,465 women were eligible for analysis, 2,992 women were TPOAb-positive, and 543 (15.67%) were TPOAb-negative. Compared with the TPOAb-negative group, the median age of the TPOAb-positive group was significantly older (33.0 vs 32.0) (*P* = 0.008), and the median serum TSH levels were significantly higher (1.56 vs 1.40) (*P* = 0.0003). However, FT4, AMH, bFSH, and bE2 levels did not demonstrate any difference between the two groups. Multivariable median regression models showed that different TPOAb titers had no effect on serum AMH, bFSH, and bE2 levels. Age-stratified analysis showed no association of TPOAb positivity with AMH, bFSH, and bE2 levels in different age groups.

**Conclusion:**

Our study indicates that TPOAb positivity is highly unlikely to impair the ovarian reserve in euthyroid women of reproductive age. A larger prospective cohort study on population should be conducted to determine this issue.

## Introduction

Thyroid peroxidase (TPO) is a low-glycosylated, membrane-bound enzyme that plays a crucial role in thyroid hormone synthesis. It is responsible for the oxidation of iodine (I_2_) and the iodination of tyrosyl residues on thyroglobulin, a key step in the production of thyroid hormones (1). TPO antibodies (TPOAb) are autoantibodies that target TPO and may act as competitive inhibitors of TPO’s enzymatic activity (2). TPOAb positivity is one of the most common biomarkers for autoimmune thyroid diseases (AITD), with 99.3% of patients with Hashimoto’s thyroiditis (HT) testing positive for TPOAb (3). The American Thyroid Association (ATA) recommends the use of TPOAb levels in scientific studies to assess the prevalence and progression of AITD (4). The prevalence of TPOAb positivity is higher in women of childbearing age, with a reported rate of approximately 14.2%, and this prevalence tends to increase with age (5, 6). Overt hypothyroidism and hyperthyroidism are known to cause pregnancy adversity and fetal morbidity. Subclinical thyroid disorders and AITD may have unfavorable effects (4).

Ovarian reserve reflects the total reproductive potential of a woman. A decline in ovarian reserve indicates a reduction in the ovaries’ reproductive and endocrine function. Anti-Müllerian hormone (AMH), basal follicle-stimulating hormone (bFSH), and estradiol (bE2), and ultrasound imaging of the ovaries to detect antral follicle count are commonly used as markers of ovarian reserve. Polycystic ovary syndrome (PCOS) is one of the most common endocrine disorders in women of reproductive age. Due to the increased follicular count, significantly elevated AMH levels are commonly observed in women with PCOS (7). Overall, AITD is not more prevalent in women with PCOS than in the general population (8). Premature ovarian insufficiency (POI) is an uncommon reproductive endocrine disorder characterized by the cessation of ovarian function before the age of 40 (bFSH >25 IU/L on two occasions over 4 weeks apart) (9). Diminished ovarian reserve (DOR) (age <40 years; bFSH >10 IU/L and AMH <1.1 ng/mL) describes women of reproductive age having regular menses but exhibiting a reduced response to ovarian stimulation compared to their peers (10), which affects approximately 26% of the infertile population (11).

POI is closely associated with autoimmune diseases, among which AITD is considered the most prevalent, reported in 14–27% of POI cases (12, 13). However, the relationship between thyroid autoimmunity and the risk of DOR or POI remains inconsistent across studies. In addition, the underlying mechanisms by which thyroid autoimmunity impacts ovarian reserve are not yet fully understood.

The reasons for the contradictory findings include differences in the studied populations, variations in the markers used to assess ovarian reserve, discrepancies in the thyroid function status of participants, and small sample sizes. For instance, a study conducted by Polyzos *et al.* in Brussels, Belgium, found no significant association between AITD or hypothyroidism and low anti-Müllerian hormone (AMH) levels (14). In contrast, Chen *et al.* reported a higher prevalence of TPOAb positivity among infertile Chinese women with low AMH levels (15). The study conducted by Hsieh *et al.* demonstrated that patients with Hashimoto’s disease had a statistically significant higher risk of amenorrhea and infertility due to ovarian failure compared to the non-Hashimoto’s disease cohort (16). In addition, research by Li *et al.* indicated that while AITD was not associated with DOR, it was significantly correlated with POI in AITD patients with TSH levels exceeding 2.5 μIU/mL (17). It is important to note that the majority of these studies were conducted at the Center for Reproductive Medicine and primarily included patients seeking fertility treatment or preservation. Consequently, the findings may not be generalizable to broader populations.

Taking into account the above-mentioned data, a cross-sectional analysis was performed to evaluate the association between TPOAb and ovarian reserve (AMH, basal FSH, and estradiol as markers) in euthyroid women at a gynecological endocrinology clinic.

## Materials and methods

### Study subjects

A cross-sectional study was conducted at Guangzhou Women and Children’s Medical Center (GWCMC), a tertiary referral hospital in South China. The study protocol was approved by the ethics committee of the institute (2023-410B01). Women who visited the Gynecological Endocrinology Clinic and underwent basal sex hormone (including FSH, LH, and E2), thyroid function screening (including TOPAb, TSH, and FT4), and anti-Müllerian hormone (AMH) tests between May 2016 and April 2024 were retrieved from the electronic medical record system. All those women were Han Chinese from South China and complained mainly of abnormal menstruation or infertility, or women preparing for pregnancy without any abnormalities requiring a hormone assessment. All participants provided written informed consent for the use of their results in research.

The study included women aged 18–45 years who attended the gynecological endocrinology clinic for menstrual abnormalities or pre-pregnancy evaluation and underwent same-day measurements of AMH, TPOAb, TSH, FT4, and basal sex hormones at our hospital’s endocrinology laboratory. All the included women did not take levothyroxine, nor did they smoke or take drugs. Exclusion criteria comprised: i) diagnoses of chromosomal abnormalities (e.g., fragile X syndrome), thyroid disorders (e.g., thyroid cancer, thyroidectomy, hypothyroidism/hyperthyroidism, or subclinical variants), ovarian cancer, or prior treatments (e.g., ovariectomy, chemotherapy, or pelvic/brain radiotherapy); ii) pregnancy; and iii) TSH or FT4 levels outside the normal reference range.

Diagnosis of POI was based on the European Society of Human Reproduction and Embryology (ESHRE) guidelines: i) oligomenorrhea or amenorrhea for at least 4 months before 40 years of age and ii) an elevated follicle-stimulating hormone (FSH) level >25 IU L^−1^ on two occasions >4 weeks apart (13). Diagnosis of PCOS was based on the 2003 Rotterdam ESHRE/ASRM PCOS Consensus Workshop Group diagnostic criteria: presenting at least two of the following criteria: i) clinical and/or biochemical hyperandrogenism, ii) oligomenorrhea or anovulation, and iii) polycystic ovaries on ultrasound (18).

### Hormone and TPOAb measurement

Serum anti-Müllerian hormone (AMH) levels were assessed by the enzyme-linked immunosorbent assay (ELISA) method using Kangrun Biotec (China) assay. Blood was drawn into plain serum tubes, centrifugation was performed within 1 h, and the serum was separated and immediately stored at 4°C and tested within 8 h. The intra- and inter-assay coefficients of variation were less than 10 and 15%, respectively. The AMH values below the detection limit (0.01 ng/mL) were calculated as 0.01 ng/mL in the statistical analysis.

The serum TSH, FT4, TPOAb levels, and basal sex hormones, including bFSH, bLH, and bE2 sampling on days 2–4 of the menstrual cycle, were measured using automatic chemiluminescence immunoassay (I2000-SR, Abbott, America). Reference ranges were as follows: TSH, 0.3–4.3 mIU/L; FT4, 12.06–23 pmol/L; and TPOAb negativity, <5.61 IU/mL. TPOAb positivity was defined as a titer ≥5.61 IU/mL.

### Statistical analysis

Patient characteristics were compared between TPOAb-positive and TPOAb-negative groups using the chi-square test. Ovarian reserve markers and thyroid function were compared using the Mann–Whitney U test, with effect sizes reported as rank–biserial correlation coefficients (*r*). Stratified analyses were performed to evaluate the impact of TPOAb titer on ovarian reserve markers (AMH, bFSH, and bE2) using multivariable median regression models with age adjustment. The association between TPOAb positivity and ovarian reserve markers was further examined using univariable median regression models stratified by age. All analyses were performed using R software (version 4.4.0).

## Results

**Figure 1:**
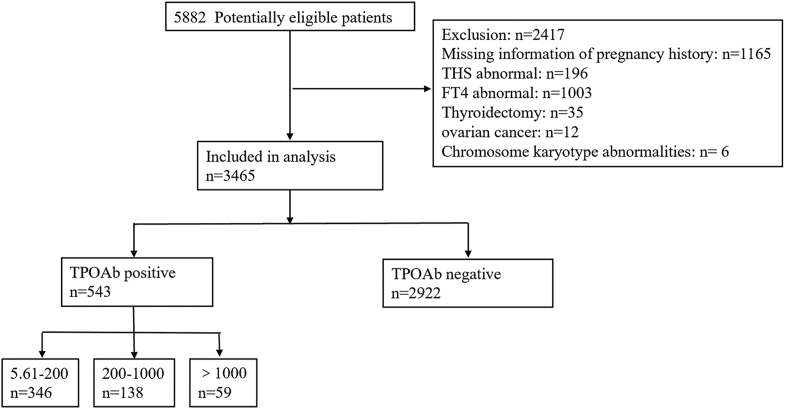
Flowchart of the study.

A total of 5,882 consecutive Han Chinese women from South China attending GWCMC between 2016 and 2024 were screened for eligibility. Of these, 3,465 met the inclusion criteria, including 2,992 TPOAb-negative and 543 TPOAb-positive women (Fig. 1).

The overall TPOAb positivity rate was 15.67%. However, the rate was significantly higher in women with a history of pregnancy (16.58%) compared to those without (13.99%; *P* = 0.0457). Three hundred twenty-one women were PCOS cases and 74 were POI cases. TPOAb positivity rates did not differ significantly between women with PCOS (14.02%, *P* = 0.3926) or POI (14.89%, *P* = 0.8827) (Table 1).

**Table 1:** Patients’ characteristics according to TPOAb.

	Total	TPOAb		
		Positive (≥5.61 IU/mL)	Negative (<5.61 IU/mL)	*P* *
Total	3,465	543 (15.67%)	2,922 (84.33%)	
Pregnancy history				0.0457
Yes	2,250 (64.94%)	373 (16.58%)	1,877 (83.42%)	
No	1,215 (35.06%)	170 (13.99%)	1,045 (86.01%)	
PCOS				0.3926
Yes	321 (9.26%)	45 (14.02%)	276 (85.98%)	
No	3,144 (90.74%)	498 (15.84%)	2,646 (84.16%)	
POI				0.8827
Yes	47 (1.36%)	7 (14.89%)	40 (85.11%)	
No	3,418 (98.64%)	536 (15.68%)	2,882 (84.32%)	

* Pearson chi-square test.

Thyroid function and ovarian reserve parameters stratified by TPOAb status are showed in Table 2. Compared to the TPOAb-negative group, the TPOAb-positive group had a significantly higher median age (33.0 vs 32.0 years; *P* = 0.008) and serum TSH levels (1.56 vs 1.40 mIU/L; *P* = 0.0003). In contrast, no significant differences were observed in FT4 (13.1 vs 13.1 pmol/L; *P* = 0.8.6), AMH (3.35 vs 3.51 ng/mL; *P* = 0.139), bFSH (5.50 vs 5.61 mIU/L; *P* = 0.281), or bE2 (150 vs 148 pmol/L; *P* = 0.792) levels between the two groups.

**Table 2:** Thyroid function and ovarian reserve of patients.

	Overall	Negative (<5.61 IU/mL)	Positive (≥5.61 IU/mL)	*P* *	Effect size
	(*n* = 3,465)	(*n* = 2,922)	(*n* = 543)		
Age (years), median (IQR)	32.0 (28.0–37.0)	32.0 (28.0–37.0)	33.0 (28.0–37.0)	0.008	−0.0714
TSH (mIU/L), median (IQR)	1.42 (1.01–1.99)	1.40 (0.996–1.96)	1.56 (1.07–2.22)	0.0003	−0.0987
FT4 (pmol/L), median (IQR)	13.1 (12.2–14.0)	13.1 (12.2–14.0)	13.1 (12.2–14.0)	0.806	0.0291
AMH (ng/mL), median (IQR)	3.48 (1.46–6.78)	3.51 (1.51–6.88)	3.35 (1.22–6.17)	0.139	0.0066
FSH (mIU/mL), median (IQR)	5.60 (4.46–6.94)	5.61 (4.48–6.96)	5.50 (4.36–6.76)	0.281	0.0399
E2 (pmol/L), median (IQR)	148 (104–248)	148 (103–247)	150 (104–260)	0.792	−0.0071

* Mann–Whitney U test.

Multivariable median regression models were used to analyze the effects of TPOAb titer on ovarian reserve with age adjustment (Table 3). First, TPOAb-positive patients were stratified into three groups based on TPOAb titer: 5.61–199 IU/mL, 200–999 IU/mL, and ≥1,000 IU/mL (Table 3). Compared to the TPOAb-negative group, none of the TPOAb-positive groups showed significant differences in serum AMH, bFSH, or bE2 levels (Table 3). Univariable median regression models were used to evaluate the impact of TPOAb positivity on ovarian reserve. Patients were stratified into five age groups: 18–24.9, 25–29.9, 30–34.9, 35–39.9, and 40–45 years (Table 4). No significant associations were observed between TPOAb positivity and AMH, bFSH, or bE2 levels across any age group (Table 4).

**Table 3:** Effects of TPOAb (categorical) on AMH, FSH, and E2, stratified by TPOAb titer.

	AMH		FSH		E2	
	Coefficient (95%CI)	*P*	Coefficient (95%CI)	*P*	Coefficient (95%CI)	*P*
TPOAb 5.61–199 vs TPOAb <5.61	−0.22 (−0.65–0.21)	0.31523	0.262 (−0.05–0.57)	0.09584	−4.90909 (−14.9–5.10)	0.33683
TPOAb 200–999 vs TPOAb <5.61	0.14 (−0.53–0.84)	0.68427	−0.116 (−0.50–0.27)	0.56149	−5.81818 (−23.01–11.37)	0.50729
TPOAb ≥1,000 vs TPOAb <5.61	0.41 (−0.94–1.76)	0.55151	−0.033 (−0.68–0.62)	0.92125	11.63636 (−8.40–31.67)	0.2551

**Table 4:** Effects of TPOAb (continuous) on AMH, FSH, and E2 stratified by age.

Age stratum	AMH		FSH		E2	
	Coefficient (95%CI)	*P*	Coefficient (95%CI)	*P*	Coefficient (95%CI)	*P*
18–24.9 (*n* = 298)*	0.00129 (−0.0047–0.0073)	0.67515	−0.00024 (−0.0015–0.0011)	0.72614	0.0055 (−0.0989–0.1099)	0.91782
25–29.9 (*n* = 921)*	−0.00068 (−0.0031–0.0017)	0.5864	−0.00007 (−0.0009–0.0007)	0.87002	0.01301 (−0.0315–0.0575)	0.56698
30–34.9 (*n* = 1,003)*	0.00064 (−0.0014–0.0026)	0.53724	−0.00025 (−0.0011–0.0005)	0.53321	−0.01601 (−0.0867–0.0547)	0.65742
35–39.9 (*n* = 783)*	−0.0001 (−0.0012–0.0009)	0.78135	0.00037 (−0.0006–0.0014)	0.47255	−0.00909 (−0.0601–0.0420)	0.72734
40–45 (*n* = 460)*	0.00008 (−0.0027–0.0004)	0.68204	−0.00109 (−0.0039–0.0018)	0.46098	0 (−0.0958–0.0958)	1

* TPOAb ≥5.61 vs TPOAb <5.61.

## Discussion

The present study was conducted in a gynecological endocrinology clinic to evaluate the impact of TPOAb positivity on ovarian reserve in euthyroid women. Our findings demonstrate that TPOAb positivity is not associated with ovarian reserve in euthyroid women, regardless of menstrual regularity. These results enhance our understanding of the relationship between AITD and ovarian function and may have important implications for fertility counseling.

AITD is the most common autoimmune disorder among reproductive-aged women. In our study population of women visiting the gynecological endocrinology clinic for menstrual abnormalities or pre-pregnancy evaluation, the TPOAb positivity rate was 15.67%, slightly higher than the previously reported 14.92%. AITD is recognized as one of the most frequent coexisting autoimmune conditions, accounting for 12–33% of POI cases (19, 20). A meta-analysis also showed that the positive rate of TPOAb was significantly increased in patients with POI and DOR, but those studies did not rule out the impact of thyroid dysfunction on ovarian reserve (21). Our data did not reveal a significantly higher TPOAb positivity rate in POI euthyroid patients compared to non-POI women (14.89 vs 15.68%). Similarly, no association was observed between PCOS and TPOAb positivity. Both POI and PCOS are conditions with distinct ovarian functional characteristics, and by including these patients, our study provides a more comprehensive investigation of this issue.

AITD exhibits a marked predilection for females and is more prevalent among individuals aged 30–50 years. Compared with nulliparous women, those who have given birth are at a relatively higher risk for AITD, particularly during the 1-year postpartum period, which is associated with an increased risk of Hashimoto thyroiditis (22, 23, 24, 25). Fetal cells can cross the placenta and enter the maternal circulation, rendering the mother microchimeric (26, 27). Fetal microchimerism has been hypothesized to be detrimental to the thyroid gland and may contribute to the pathogenesis of AITD (28, 29). In the present study, we found that the positive rates of TPOAb in patients with a history of childbirth were slightly higher than those without childbirth, although the difference was not statistically significant. However, the positive rates of TPOAb in patients with a history of pregnancy were significantly higher than those without pregnancy. Our results suggest that fetal microchimerism might affect the thyroid gland from the early stages of pregnancy, rather than solely from the postpartum period.

Thyroid autoantibodies have been detected in the follicular fluid of women with thyroid autoimmunity, with a strong correlation observed between serum and follicular fluid antibody levels (30, 31). This suggests that TPOAb can translocate from plasma to follicular fluid. However, the underlying mechanisms by which thyroid autoantibodies may affect ovarian function remain unexplored in basic experimental studies. In clinical studies, which predominantly include infertile women visiting reproductive medicine centers, findings on the association between serum TPOAb levels and ovarian reserve remain inconsistent.

Our findings revealed significantly higher serum TSH levels in TPOAb-positive women compared to their TPOAb-negative counterparts. However, no significant differences were observed in FT4, AMH, basal FSH, or E2 levels between the two groups. These results indicate that while TPOAb positivity correlates with elevated TSH levels, it does not appear to significantly affect ovarian reserve biomarkers. Stratified analyses adjusting for age-related declines in ovarian reserve further demonstrated no dose-dependent relationship between TPOAb titers (categorized as 5.61–199 IU/mL, 200–999 IU/mL, and ≥1,000 IU/mL) and ovarian reserve markers. Furthermore, age-stratified evaluations across five reproductive age cohorts (18–24.9, 25–29.9, 30–34.9, 35–39.9, and 40–45 years) consistently demonstrated no significant association between TPOAb status and ovarian reserve parameters.

While our findings contrast with two prior studies (Saglam *et al.* (32) and Magri *et al.* (33), reporting lower AMH levels in 85 and 55 European women with AITD compared to 82 and 233 controls, respectively), they align with five other investigations. Unuane *et al.* (34) found no AMH differences between TPOAb/TGAb-positive and negative euthyroid patients. Similarly, Li *et al.* (17) demonstrated no TPOAb-AMH correlation in women aged 18–40 (excluding PCOS cases) with normal/low ovarian reserve. Three additional small-sample studies also reported comparable AMH levels between TPOAb-positive and negative women (35, 36, 37). Notably, unlike previous reports focusing on infertile populations, our study specifically evaluated women presenting to gynecological endocrinology clinics for menstrual abnormalities or pre-pregnancy assessments.

The key strengths of this study include: i) a large sample size (*n* = 3,465) powered for cross-sectional analyses, and ii) inclusion of both reproductively normal women and those with menstrual abnormalities, representing a clinically relevant population distinct from previous infertility-focused cohorts. However, several limitations must be acknowledged. First, the cross-sectional design introduces potential selection bias and unmeasured confounding. Second, the lack of body mass index data may compromise adjustment for metabolic confounding factors. Third, this study failed to investigate the combined impact of TGAb positivity and other autoimmune diseases on ovarian reserve. Future investigations should validate these findings through prospective population-based cohorts with comprehensive metabolic profiling and other autoimmune diseases.

## Conclusion

This large-scale study provides robust evidence that TPOAb positivity does not impair ovarian reserve biomarkers (AMH, bFSH, bE2) in euthyroid reproductive-aged women, regardless of menstrual regularity. While confirming the association between TPOAb and subclinical TSH elevation, our findings challenge the clinical relevance of thyroid autoimmunity in ovarian aging. Future research should prioritize mechanistic studies to elucidate thyroid–ovarian axis interactions in AITD.

## Declaration of interest

The authors declare that there is no conflict of interest that could be perceived as prejudicing the impartiality of the work reported.

## Funding

The study was supported by a grant from the Guangdong Basic and Applied Basic Research Foundationhttps://doi.org/10.13039/501100021171 Committee, Guangdong, China (2023A1515220201). The funder had no role in study design, data collection and analysis, decision to publish, or preparation of the manuscript.

## Author contribution statement

Chongjuan Gu and Yaojuan He contributed to the design of the research and drafted the manuscript. Chaomin Yue and Jie Li contributed to data management and prepared the retrospective data for analysis. Ling Li, Chongjuan Gu, and Chunting Wu contributed to the development of the statistical plan and statistical analysis of the data. All authors reviewed the manuscript and approved the final version.

## Data availability

The datasets generated during and/or analyzed during the current study are available from the corresponding author on reasonable request.

## Ethics statement

This study follows the ethical guidelines of the Helsinki Declaration and was approved by the Ethical Committee of the Guangzhou Women and Children’s Medical Center (2023-410B01). The patients provided written informed consent for clinical information to be reported in the journal.
